# Outcome Study of Mandibular Fractures Treated by Surgical Stabilization With Plates and Screws

**DOI:** 10.7759/cureus.58561

**Published:** 2024-04-18

**Authors:** Abhishek Rai, Vinay Karwal, Shuchi Nigam, Atul Saxena, Manish Sharma

**Affiliations:** 1 Burn and Plastic Surgery, All India Institute of Medical Sciences, Deoghar, Jharkhand, IND; 2 Plastic and Reconstructive Surgery, Adesh Medical College and Hospital, Kurukshetra, IND; 3 Anesthesiology and Critical Care, Uttar Pradesh University of Medical Sciences, Etawah, IND; 4 Plastic and Reconstructive Surgery, Uttar Pradesh University of Medical Sciences, Etawah, IND; 5 Plastic and Reconstructive Surgery, Pushpanjali Hospital, Agra, IND

**Keywords:** occlusal alignment, screws, plates, surgical stabilization, mandibular fracture

## Abstract

This study aims to assess the outcomes of mandibular fractures treated through surgical stabilization using plates and screws, focusing on factors such as postoperative complications, patient satisfaction, and functional recovery. A total of 42 patients were included in the study. Surgical interventions involved the application of plates and screws at the fracture sites. Postoperative complications, including infection, hardware failure, and malocclusion, were recorded. Surgical stabilization of mandibular fractures using plates and screws demonstrates favorable outcomes in terms of stability, occlusal alignment, and patient satisfaction. The findings of this study contribute valuable insights into the efficacy of this surgical approach, highlighting its role in achieving successful outcomes for mandibular fracture management. Further prospective studies and randomized controlled trials are recommended to strengthen the evidence base and refine treatment protocols.

## Introduction

Facial fractures are one of the most common injuries to the facial area. Mandibular fractures are the second most common bone fracture in the maxillofacial area after the nasal bone. Because the mandibular bone is the most movable and prominent in the facial area, the number of mandibular fractures far exceeds the number of zygomatic fractures and maxillary fractures. The ratio of mandibular to zygomatic and maxillary fractures is 6:2:1 [1]. Because mandibular fractures can affect function and appearance, they need to be diagnosed and treated appropriately.

Maxillofacial fractures, in particular mandibular fractures, are frequently caused by a variety of traumas [2]. Several environmental, cultural, and socioeconomic factors contribute to the reported diversity of their incidence and etiology [3-6]. The primary causes in developed nations seem to be falls, assaults, traffic accidents, and sports-related injuries [5,7-9]; however, in less developed economies, interpersonal violence is typically the cause [5,10]. The literature recommends a variety of methods for managing mandibular fractures, including the use of plates, screws, bandages, external appliances, extraoral and intraoral appliances, monomaxillary wiring, and intermaxillary wiring. Miniplate fixation along the *ideal lines of osteosynthesis* has become the most popular technique among the different treatment modalities available. Numerous clinical studies have shown the potential and efficacy of the Champy miniplate since its introduction in the treatment of mandibular fractures [3]. Theoretically, rigid immobilization creates an optimal environment for osteogenesis to occur without interfering with mobility at the fracture site [11]. The mandibular fractures are subject to strong biomechanical forces and are highly susceptible to salivary contamination. Therefore, the choice of a particularly stable fixation for their treatment is determined by these two factors.

Mandibular fractures typically involve multiple systems, necessitating management of breathing, circulation, and airway patency. As a result, coordination between various medical science disciplines is required. It is also necessary to rule out any additional serious injuries. These may take the form of radiological investigations or clinical assessments. Mandibular fractures can be problematic, but they are rarely fatal. They should therefore be handled following initial resuscitation, patient stabilization, and the treatment of any injuries that pose a threat to life. Therefore, appropriate triage is required.

This study aims to evaluate the stability of mandibular fractures treated by surgical stabilization and the postoperative occlusion after plate and screw fixation for mandibular fractures.

## Materials and methods

This observational study was carried out after receiving approval from the institutional ethical committee of R. G. Kar Medical College (Ethical clearance no. RKC/Ethica/45-16/02/18), and all the procedures were carried out in compliance with the 2013 Helsinki Declaration for 18 months from April 2018 to October 2019. This study was conducted in the Department of Plastic and Reconstructive Surgery of R. G. Kar Medical College, Kolkata, India.

A total of 42 patients with isolated mandibular fractures of less than one-week duration admitted to the Department of Plastic Surgery of our institute were included in the study. Patients who refused to participate in the study, those with compound fractures, other facial bone fractures, or associated head injuries were excluded from the study.

We had planned for the definitive treatment of mandibular fractures after stabilizing the patient's condition and excluding the possibility of additional injuries. The fracture site was exposed by either an intraoral or extraoral route following intermaxillary fixation. Two plates and screws were used to fix the fracture after it had been reduced. Patients were instructed to perform mouth-opening exercises after surgery. The arch bar was taken out once their mouth opened sufficiently (more than three fingers).

Surgical technique

Each case was performed with the patient in a supine position under general anesthesia with nasotracheal intubation. The approach of the lower gingivobuccal sulcus was used to reveal the fractures of the body, symphysis, and parasymphysis. To expose the fracture site to the lower border of the mandible, a mucoperiosteal flap was raised and a low-level vestibular incision was made close to the fracture site. Extreme caution was used to avoid damaging the mental nerve. Angle fractures were treated using the Risdon method. It was determined that the mandibular marginal nerve needed protection. 

**Figure 1:**
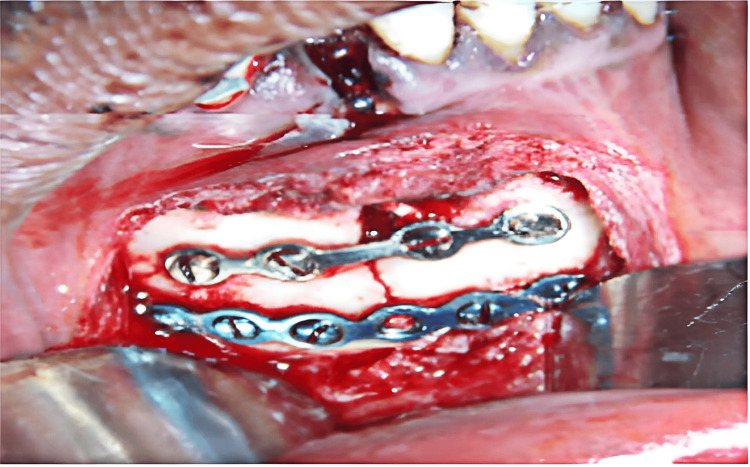
Mandible fixation with standard mini plates.

For fixation, we used 2 mm x 8 mm screws and standard titanium mini plates measuring 2 mm (Figure 1). In accordance with Champy's line of osteosynthesis, 2 four-hole conventional mini plates were utilized for the symphysis and parasymphysis fractures between the mental foramina.

The course of a tension line at the base of the alveolar process and an additional line to counteract twisting forces near the lower border of the jaw are aligned with these lines. 

The two plates were kept parallel and at a distance of 4 to 5 mm. Using 2 mm x 8 mm screws on either side of the fracture, the upper plate was fixed first, followed by the lower plate. After verifying the occlusion, the screws were lastly tightened. The fracture site was irrigated, and closure was done in two layers with 2-0 Vicryl. For five days, all patients were kept under antibiotic cover. In cases where two mini plates were used, maxillomandibular fixation (MMF) was removed soon after the surgery. In cases where a single plate was used, MMF was retained for two weeks.

After the second postoperative week, they began mild physical therapy. For the first six weeks, follow-up was done every week; after that, it was done every three months.

## Results

We studied a total of 42 patients with mandibular fractures with ages ranging from 11 to 56 years, with a mean age of 30.1 years, of which 38 (90.48%) were male and 4 (9.52%) were female, with a male-female ratio of 9.5:1 (90.48% male and 9.52% female) (Table 1). The reasons for this discrepancy between sexes were probably due to rash driving, occupational hazards, substance abuse, as well as interpersonal violence.

**Table 1:** Male and female patients distribution.

S.No.	Sex	Number of patients, *n* (%)
1	Male	38 (90.48% )
2	Female	4 (9.52%)

We observed the commonest cause of injury to be road traffic accidents (31, 73.81%), followed by falls from height (3, 7.14%), assault injuries (7, 16.67%), and fall of heavy objects(1, 2.38%) (Table 2). 

**Table 2:** Cause of injury.

No.	Cause of Injury	Number of patients, *n* (%)
1.	Road traffic accident	31 (73.81%)
2.	Falls from height	3 (7.14%)
3.	Assault injuries	7 (16.67%)
4.	Fall of heavy objects	1 (2.38%)

Out of the 42 patients, 26 (61.90%) patients had a single fracture, 14 (33.33%) patients had two fractures, and 2 (4.76%) had a triple fracture of the mandible.

**Figure 2:**
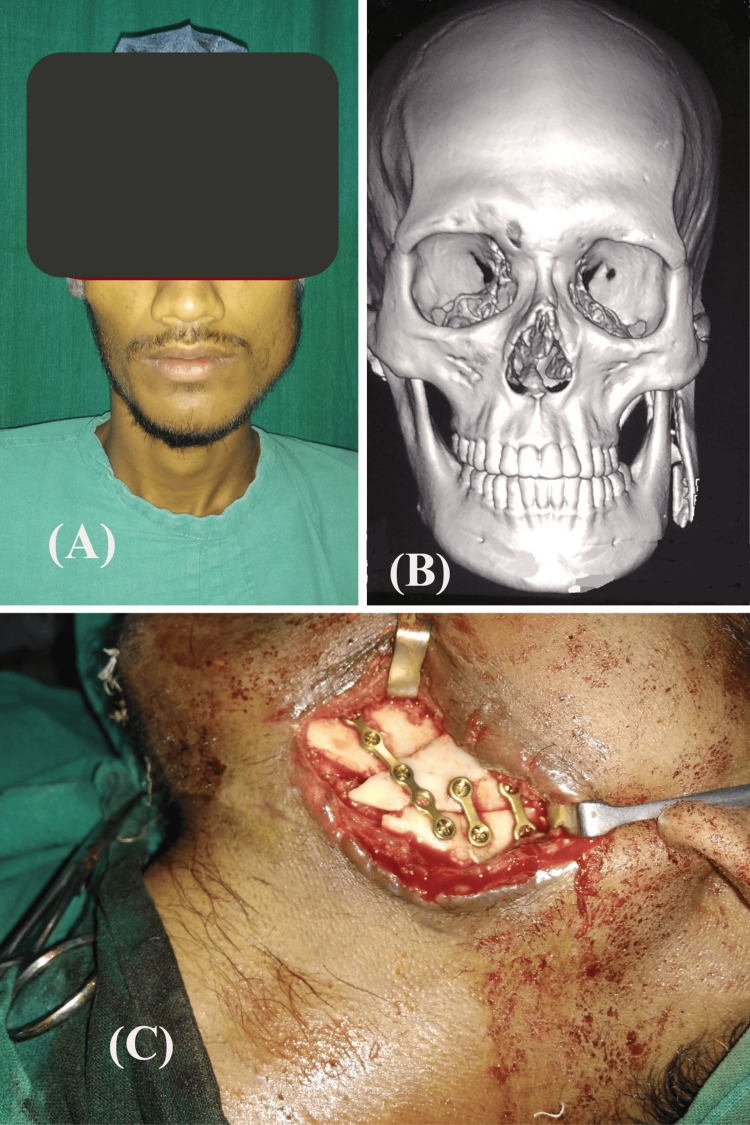
Fracture angle of the mandible: (A) preoperative picture of the patient; (B) CT scan showing the fracture; and (C) fracture stabilization with mini plates.

Out of 42 patients, 17 (40.48%) had suffered a fracture at the parasymphysis, 6 (14.29%) had a fracture at the body, 3 (7.14%) had a fracture at the angle (Figure 2), 9 (21.43%) had a fracture at bilateral parasymphysis, 5 (11.90%) had a fracture at both the parasymphysis and body, and 2 (4.76%) had suffered a fracture at both the parasymphysis and angle of the body (Table 3). The right side of the mandible is fractured more commonly than the left side. The total number of patients with right-sided mandibular fractures was 25 (59.52%), compared to 17 (40.48%) patients with left-sided mandibular fractures.

**Table 3:** Distribution of patients according to the site of injury.

No.	Site of injury	Number of patients, *n* (%)
1.	Parasymphysis	17 (40.48%)
2.	Body	6 (14.29%)
3.	Angle	3 (7.14%)
4.	Bilateral parasymphysis	9 (21.43%)
5.	Parasymphysis and body	5 (11.90% )
6.	Parasymphysis and angle	2 (4.76%)

The average number of days between injury and surgical management was six days. The minimum number of days was two, while the maximum was 14 days. The main reason behind the delayed surgical procedure was the late presentation of the patient to our department.

For the management of open reduction and titanium plating, we utilized two-hole plates with a 2 mm gap, four-hole plates with a 2 mm gap, six-hole plates with a 2 mm gap, four-hole plates without a gap (2 mm), and six-hole plates without a gap (2 mm). We observed similar results and outcomes with the various types of plates described above.

Angle Class I occlusion was achieved in all the patients (42). Postoperatively, three patients had surgical site infections and three patients had neurological deficits. The neurological deficit improves with time. In all the patients, mouth opening of >3 fingers was achieved with normal occlusion on follow-up. The period of hospital stay varies from four to 10 days. The average hospital stay was 6.14 days. Infection was responsible for the greater number of hospital stays.

## Discussion

The mandible, which is regarded as the strongest and heaviest facial bone, is more vulnerable to fractures due to its open arch shape, lower facial location, and aging process. Fractures can result from facial injuries, which harm the bone in addition to soft tissues. Strong muscles connect the mandible to perform a variety of tasks. Though these strong muscles can cause massive displacement of the fracture fragments, they also serve as a splint and protect the mandible.

Since the face often serves as the initial point of contact in social situations, injuries and mutilations to the face can have a catastrophic effect on the victim. Therefore, understanding the dentition is a crucial requirement for treating mandible fractures appropriately. If mandibular fractures are not properly treated, malocclusion is inevitably the result.

In our study, injuries from traffic accidents accounted for the majority (31, 73.81%), with falls from height and assault coming in second and third, respectively. According to Adekeye, 74% of mandibular fractures were caused by auto accidents [12]. Subhashraj et al. also reported this in a study conducted in a city in South India [13]. An average of 1.45 fractures per person was observed, with 61.9% of single mandibular fractures and 38.10% of multiple mandibular fractures. The findings of Sirimaharaj and Pyungtanasup [14], who reported 1.4 fractures per person, are comparable to this.

Every case of parasymphysis and body fracture was treated intraorally. Angle fractures were treated with an extraoral technique. When using the Risdon approach, caution was taken to avoid damaging the marginal mandibular nerve or the mental nerve.

During the postoperative phase, there were three cases of infection (7.14%), which were managed with higher antibiotics and frequent dressings. None of the patients needed implant removal. The infection rate was similar to 8.1%, as reported by Oginni et al. [15]. Three (7.14%) patients had a neurological deficit, which was similar to the findings of study results by Benjamin et al. [16] and Cawood [17], which showed improvements in six to eight weeks for both groups. This deficiency was caused by the nature of the injury rather than the surgical procedure.

The time interval between the surgical procedure and the injury was, on average, six days. However, it varied from two to 14 days. The patient's delayed arrival at our department was the primary cause of the surgical procedure's delay.

On average, 6.14 days were spent in the hospital. The duration varied from four to 10 days. Longer stays in the hospital were caused by infections.

A limited sample size (42 patients), a brief follow-up period that may have missed late problems, and selection bias resulting from the single-center analysis were among the limitations of this study. Larger cohorts, more varied cases, and longer follow-up periods in future studies may improve comprehension of the effects of this treatment and guide therapeutic practice.

## Conclusions

Males make up the majority of patients with mandibular fractures, and they tend to be younger. Most mandibular fractures occur as a result of road traffic accidents. Trauma to the canine and premolar, angle, and molar regions of the jaw most frequently results in mandibular fractures. The results of treatment are unaffected by the cause of the injury. The type and site of fracture only slightly affect how well treatment goes. To treat mandibular fractures, a closed or open technique must provide sufficient fracture reduction and stabilization. The restoration of a healthy dental occlusion and bony union is necessary for success.

While intraosseous wiring reduced distraction, it did not result in long-term inter-fragmentary compression. Due to this, internal fixation with mini plates and open reduction are becoming more and more popular. As a result, patients are now able to return to work earlier and experience less weight loss, reduced malocclusion, nonunion, and improved mouth opening and speech.
